# Modeling the Burden of Extreme Weather Events in a Large Network of International HIV Care Cohorts

**DOI:** 10.1029/2025GH001514

**Published:** 2025-11-03

**Authors:** Sophia D. Arabadjis, Frank Davenport, Ana Maria Vecedo Cabrera, Zachary Shahn, Ellen Brazier, Andrew Maroko, Avantika Srivastava, Gad Murenzi, Timothy John Dizon, Keri N. Althoff, Antoine Jaquet, Aggrey S. Semeere, Yanink Caro Vega, Mark K. U. Pasayan, Sheri D. Weiser, Denis Nash

**Affiliations:** ^1^ Institute for Implementation Science in Population Health City University of New York Graduate School of Public Health and Health Policy New York NY USA; ^2^ University of California Center for Climate Health Equity University of California San Francisco San Francisco CA USA; ^3^ Climate Hazards Center Department of Geography University of California Santa Barbara CA USA; ^4^ Institute of Social and Preventive Medicine University of Bern Bern Switzerland; ^5^ Oeschger Center for Climate Change Research University of Bern Bern Switzerland; ^6^ Icahn School of Medicine at Mount Sinai Institute for Health Equity Research New York City NY USA; ^7^ Department of Epidemiology and Biostatistics City University of New York New York City NY USA; ^8^ Research for Development and Rwanda Military Referral and Teaching Hospital Kigali Rwanda; ^9^ Department of Health Research Institute for Tropical Medicine Muntinlupa Philippines; ^10^ Department of Epidemiology Johns Hopkins Bloomberg School of Public Health Baltimore MD USA; ^11^ Bordeaux Population Health Centre National Institute for Health and Medical Research (INSERM) UMR 1219 Research Institute for Sustainable Development (IRD) EMR 271 University of Bordeaux Bordeaux France; ^12^ Infectious Diseases Institute Makerere University Kampala Uganda; ^13^ Departamento de Infectología Instituto Nacional de Ciencias Médicas y Nutrición Salvador Zubirán Mexico City Mexico; ^14^ Division of HIV/AIDS San Francisco General Hospital University of California at San Francisco San Francisco CA USA

**Keywords:** HIV/AIDS, GIS, extreme weather events, drought, floods, risk

## Abstract

Extreme weather events (EWEs) continue to threaten the health and well‐being of populations across the globe. However, risk from drought and floods is not evenly distributed spatially nor are all populations equally at risk for poor health outcomes. Globally, people living with HIV/AIDS (PLHIV) face a particular set of challenges with EWE exposure including increased susceptibility to disease progression from care disruptions and medication adherence, and general population concentration in areas where rainfall is both highly variable and key to economic well‐being. To mitigate the impacts of EWE exposure on PLHIV, it is necessary to understand the historical EWE exposure patterns at HIV care clinics. In this paper, we link open‐source measures of drought and flood events to clinic locations from the International epidemiology Databases to Evaluate AIDS (IeDEA) network, a longitudinal study of over 2 million people living with and at risk for HIV in 44 different countries around the globe enrolling in HIV care from 2006 to present. Using generalized additive models fit to clinic‐level drought and flood exposures, we show how exposures vary across and within countries, model each clinic's probability of exposure to a drought or flood to identify high‐risk areas, and describe how this historical exposure record could ultimately be used to identify at‐risk populations for a wide variety of study designs. While EWEs occurred at HIV care clinics around the globe, we found that clinic locations in Southern Africa are particularly vulnerable to flood and drought events as compared to other IeDEA clinic regions and locations.

## Plain Language Summary

Extreme weather events (EWEs) threaten human health and well‐being through environmental, economic, social and health pathways such as damaging crops and livestock, destroying infrastructure, displacing populations, and creating conditions for increased transmission of diseases. Some populations, like people living with HIV (PLHIV), are particularly vulnerable to EWEs because of additional health risks and location (The global burden of HIV is concentrated in areas most effected by EWEs). To understand how EWEs impact HIV care provision and health outcomes, researchers need large cohort studies matched with long histories of EWE exposure. In this paper, we combine seasonal measures of drought and flood to establish a historical exposure record from 1981 to present for 777 HIV clinic locations in the International epidemiology Databases to Evaluate AIDS (IeDEA). We use gridded precipitation data from 1981 to 2023 to determine droughts that occur during wet months and archive‐based flood data from 2006 to 2023 to determine flood exposures for clinics and patients. We model these exposures to identify clinic locations at higher risk for flood, drought, and multi‐hazard risk. The approaches used in this study provide a guide for future researchers and identify geographic locations for place‐specific research designs to examine the effects of EWE exposures on HIV health outcomes.

## Key Points

- Open data sources can be used to establish robust historical drought and flood event records across an international network of HIV care clinics
- Modeling flood and drought exposures across a clinic network reveals clinic locations at high‐risk for exposure to drought and flood events
- This step‐by‐step methodology can be used by researchers to identify comparative exposures and causal research designs

## 1 Introduction

Extreme weather events (EWEs) such as floods and droughts present numerous threats to environmental systems essential to human health and well‐being. Both floods and droughts can disrupt healthcare infrastructure and delivery, agricultural yield leading to food and water insecurity and poor nutrition, poverty, increased displacement/migration, and steep economic losses (Alderman et al., 2012; Carroll et al., 2010; Epstein et al., 2019; Paranjothy et al., 2011; Phalkey et al., 2015; Rentschler et al., 2022; Stanke et al., 2013; van Loenhout et al., 2020). Both drought and flooding are associated with negative health outcomes for exposed populations, including, but not limited to, higher prevalence of infectious disease (Beebe et al., 2009; Bouma & Dye, 1997; Cann et al., 2013; Ding et al., 2013; Medlock & Leach, 2015; Stanke et al., 2013), low birthweight and/or pregnancy loss (Davenport et al., 2020; He et al., 2024), and general mortality (IPCC, 2022). Droughts in particular have an out‐sized effect on mortality despite their relatively low global frequency. From 1970 to 2019, less than 10% of all natural disasters were drought‐related, but they contributed to 34% of disaster‐related deaths, largely concentrated on the African continent (IPCC, 2022).

As the frequency and intensity of droughts, floods and other extreme weather events change globally, some populations are particularly vulnerable to exposure. Vulnerability can be locational (e.g., residing in a flood plain) as well as societal or social (e.g., poverty status, race, gender, governance challenges, tenuous access to basic needs, limited social capital, climate‐sensitive livelihood, or others) (Benevolenza & DeRigne, 2019; Cutter et al., 2003, 2009, 2012; IPCC, 2022). Globally, the Intergovernmental Panel on Climate Change (IPCC) highlights seven regional hotspots of both locational and social vulnerability to climatic hazards, which closely mirrors the global hotspots of the HIV pandemic and people living with HIV (PLHIV) (IPCC, 2022; Watts et al., 2015; WHO, 2024b).

In addition to social and locational vulnerabilities, the 39.9 million PLHIV face a unique set of health challenges with extreme weather exposure (WHO, 2024a), such as access to clinics for regular care and medication (Epstein et al., 2023; Iwuji et al., 2023), comorbidity, and competing risks such as diarrheal disease, vector‐borne disease and cardiopulmonary disease (Smith et al., 2014; Stanke et al., 2013). They also face social challenges related to prevailing cultural norms such as HIV‐related stigma and/or coping strategies that they may feel compelled to adopt for survival such as economic‐driven sexual behavior and migration (Orievulu, Ayeb‐Karlsson, Ngwenya, et al., 2022; Trickey et al., 2024; Weiser et al., 2007). Given these additional vulnerabilities, understanding the relationships between PLHIV's exposure to extreme weather events and their subsequent HIV care outcomes is critical for policy makers, care providers, and community members to anticipate and adapt care to changing climate.

The pathways linking EWE exposure to negative HIV care outcomes (individual level) and/or HIV care service disruption (clinic level) are often complex and place‐specific (Iwuji et al., 2023, 2024; Orievulu, Ayeb‐Karlsson, Ngema, et al., 2022; Orievulu, Ayeb‐Karlsson, Ngwenya, et al., 2022). At the individual level, while proximal injury‐driven pathways are possible, there are also many less direct (distal) pathways, often mediated by economic, social, or infrastructure constraints (Lieber et al., 2022; Orievulu, Ayeb‐Karlsson, Ngwenya, et al., 2022; Treibich et al., 2022; Trickey et al., 2024). Examples of nodes along these pathways could include increased prevalence of transactional sex to smooth income (Treibich et al., 2022), increases in vector‐borne disease and comorbidity (Suhr & Steinert, 2022), or localized lack of electricity because of water shortages and dependence on hydropower (Falchetta et al., 2019). In a review focused on extreme weather events and disruptions to HIV care specifically, Iwuji and co‐authors identified five broad themes that link EWE exposure to HIV care and care outcomes: economic and livelihood conditions, psycho‐social factors, infrastructure damage and operational challenges, migration and displacement, associated medical conditions and health care needs (Iwuji et al., 2024). In another review focused on HIV treatment adherence and drought exposure in Africa, co‐authors identified similar themes (Orievulu, Ayeb‐Karlsson, Ngema, et al., 2022). Indeed, much of the literature reviewed in these articles aims to either disentangle one or two of these pathways or simply to document changes in care‐seeking, care‐provision or health outcomes. Ultimately, the exposure methodologies described in this article represent a first step in a broader effort to unpack local impacts and effects from EWE exposure.

To facilitate research in this area, this study describes a method to determine clinic‐level exposure to two primary EWEs (flooding events and drought conditions) in an international network of HIV care clinics. The International epidemiology Databases to Evaluate AIDS (IeDEA) pools observational clinical data on more than 2.2 million PLHIV and people at risk for HIV in 44 countries in seven geographic regions from 2004 to present (IeDEA Website, 2024; Zaniewski et al., 2018). In this paper, we link global estimates of precipitation, temperature and flood events to determine exposure to drought and floods for all clinics contributing data to the IeDEA network between 2006 and 2023. Based on these exposures, we then estimate risk of drought, flood or multi‐hazard and rank clinics globally using a regression framework that allows for non‐linear space and time effects. Our aims are three‐fold: (a) to outline procedures for identifying preliminary drought and flood exposures across IeDEA clinic sites, (b) to identify high‐risk regions within the IeDEA network and (c) to guide future research of EWE and HIV outcomes using these data sources and approaches.

## 2 Materials and Methods

### 2.1 Drought

We measure seasonal drought using an established metric: the standardized precipitation‐evapotranspiration index (SPEI) (Vicente‐Serrano et al., 2010). SPEI is an enhanced version of another widely used and established drought metric, the SPI (standardized precipitation index). Like the SPI, the SPEI is simple to calculate, multiscalar (time and space independent), and easy to interpret as a z‐score of rainfall distribution based on a prespecified historical period (McKee et al., 1993, 1995). Unlike the SPI, however, the SPEI takes into account the effect of rising global temperatures on climatic water balance by incorporating evaporative demand into the calculation (Beguería et al., 2014; Vicente‐Serrano et al., 2010).

**Figure 1:**
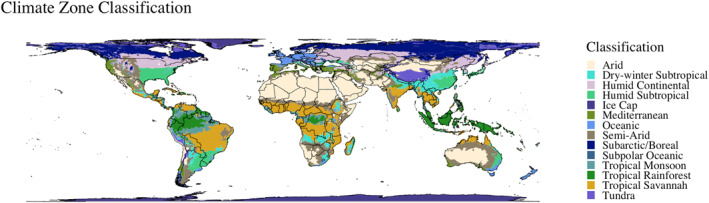
A visualization of a the simplified Köppen–Geiger climate classification system illustrates the different global climatic zones.

Evaporative demand (ET_0_) is sometimes described as the “thirst of the atmosphere,” and reflects the gradient between water availability in the atmosphere and water availability from other sources (ground water, soil moisture, surface water, reservoir storage etc.) (Vicente‐Serrano et al., 2010). A high evaporative demand indicates a higher imbalance between atmospheric conditions and land conditions (non‐linearly), and results in more evapotranspiration (Vicente‐Serrano et al., 2010). The incorporation of evaporative demand for drought monitoring is particularly important for tropical, Mediterranean and semi‐arid climates because a higher mean temperature (even if minimal), significantly increases the evaporative demand and can cause lasting water deficits (Vicente‐Serrano et al., 2010). Figure 1 shows the updated Köppen‐Geiger climate classification, slightly simplified to highlight the tropical, Mediterranean, and semi‐arid climates (Kottek et al., 2006).

From a global health perspective, seasonal drought (as measured by the SPEI) is particularly troubling if it occurs at traditionally wet times of the year (i.e., rainy seasons) as these are often periods of critical food production for agriculturalists, pastoralists, and other population groups that depend on crop and livestock production for income and/or basic subsistence. Droughts during these periods can affect regional crop cycles and, in the worst cases, result in widespread food shortages (Funk et al., 2019; Rosegrant & Cline, 2003). In this paper, we specifically focused on seasonal droughts that occurred during the three wettest months of the year, but we began by calculating SPEI for every clinic location for every month from 1981 to 2023 and then filtered to wet‐months of interest.

To calculate monthly precipitation, we used the Climate Hazards Infrared Precipitation with Stations (CHIRPS) v2 and tabulated the monthly precipitation for each clinic point location (with a 100 km buffer) for each of the 528 months from 1981 to 2023 (see Figure 3) (Funk et al., 2015). For our monthly measure of ET_0_, we relied on the National Oceanic and Atmospheric Administration's Physical Science Division (NOAA‐PSD) reference ET_0_ data set for evaporative demand (Hobbins et al., 2016; McEvoy et al., 2016). Both CHIRPS and the NOAA‐PSD ET_0_ are used globally in operational monitoring and forecasting of drought and drought impacts (Funk et al., 2019). We used the difference between precipitation and ET_0_ to estimate monthly water deficit/surplus in each location and then used the R code libraries documented in Begueria et al. (2014) to calculate the monthly SPEI (Beguería et al., 2014; Beguería & Vicente‐Serrano, 2023; Salvador et al., 2024). As the reference period in our SPEI calculation, we used a 30‐year interval from 1981 to 2012.

**Figure 2:**
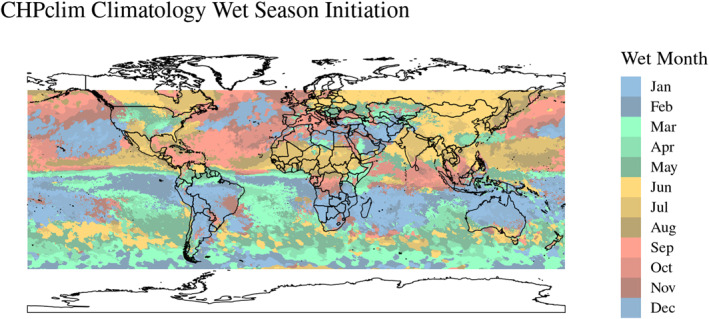
CHPclim wettest 3 months timing (wet season onset or initiation) are displayed in this graphic. The colors represent the first month of the three wettest months (based on the CHPclim climatology). For much of Australia, the wettest 3 months begin in December or January and run through February or March, respectively. Conversely much of China's three wettest months begin in June (light yellow) and run through August.

Finally, we then filtered to wet‐months using the CHPclim 2.0 global mean gridded rainfall to identify the first month of the three‐wettest months for each location (see Figure 2) (Funk et al., 2015). To account for variability of wet season onset and duration in each calendar year, we also used 1 month pre‐onset and 1 month post for a total of 5 months. SPEI values less than −1.3 were considered “drought conditions” and values less than −2 were considered “exceptional drought conditions” per the standards given by the U.S. Drought Monitor (National Drought Mitigation Center (NDMC) University of Nebraska‐Lincoln et al., 2023).

### 2.2 Flood Events

Flooding events are the most common EWE and have widespread impacts in both high income and low‐ and middle‐income countries (Kettner et al., 2021; UCLouvain, 2024). However, floods are not easily measured. Even in high resource areas where hydrologic measurements from stream or riverbeds may be available, measurements (and devices) from inundated flood plains are often not directly available (Kettner et al., 2021). Such issues are compounded by a lack of data sharing for existing measures; hydrological data from upstream may not be communicated across political boundaries in a timely (or free) way. For many years, satellite imagery has been an important source of flooding events. Advancements in optical and synthetic aperture radar sensors have allowed researchers to map flood extents with a relatively low latency period (almost real time) (Schumann et al., 2018).

We used the Global Active Archive of Large Flood Events (GAALFE) from 2006 to 2021 to determine clinic exposure to flood events (Brakenridge, 2024). Curated by the Boulder (formerly Dartmouth) Flood Observatory, GAALFE tracks, monitors and archives flood events globally, using news reports, government reports, and satellite imagery from MODIS and related earth‐observation data (Brakenridge, 2024; Schumann et al., 2018). GAALFE leverages additional information in news reports and links additional attribute data to each flood including a number of displaced persons. This number varies by event; sometimes it is the number of people affected by the flood, sometimes it is the number of people left homeless after the incident, and sometimes it is the number of people evacuated during the flood. GAALFE provides additional documentation on its systematic assignment of the number displaced (Brakenridge, 2024). In addition, each flood event has an associated geo‐referenced polygon (footprint), event start‐time and event stop‐time (estimated when displaced persons can return home.)

**Figure 3:**
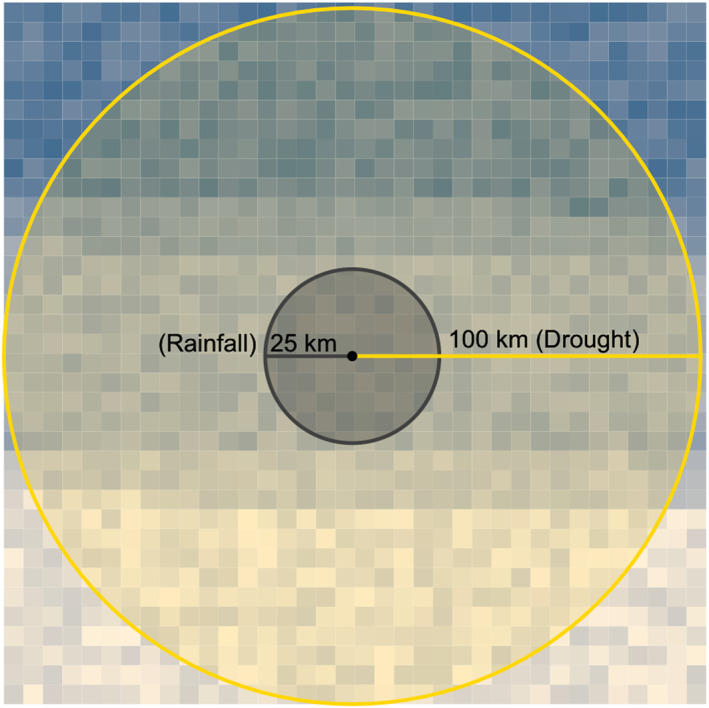
Each IeDEA network clinic is buffered by 100 km for drought creating a spatial polygon across which raster cells are averaged to calculate monthly SPEI. For rainfall during flood events, precipitation is summed for the 25 km area around each IeDEA network clinic for the duration of the flood event.

We considered IeDEA clinics “exposed” if the location of the clinic overlapped with the extent of a flood event designated by GAALFE. Using the date interval associated with each flood event, we summed the daily spatial averages of CHIRPS precipitation estimates for the buffered clinic location during the event to get an estimate of total precipitation during the exposure period (see Figure 3). We also included the duration of the flood event (in days), and the interstices flood period (number of days since the last flood event). Due to data quality concerns, we excluded floods that occurred prior to 2006 or floods with a duration of greater than 100 days and less than 3 days. Embedded in both our flood and drought exposure metrics is the assumption that any flood and/or drought exposure recorded for the clinic are also experienced by the patients attending those affected clinics. This assumption may be more valid in some areas and time periods than others; this is a point we return to in the discussion.

### 2.3 IeDEA Research Consortium

**Figure 4:**
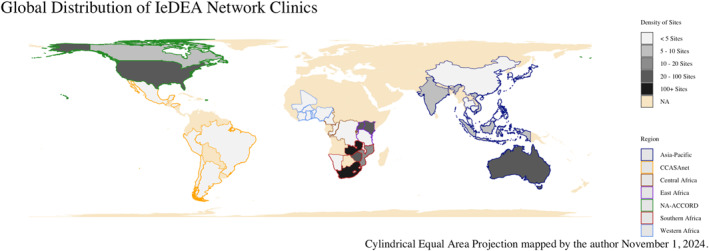
The IeDEA network clinics are widely distributed across climatologic zones and continents. The majority of clinic sites are concentrated in Sub‐Saharan Africa. The distribution of clinics largely reflects the global distribution of people living with HIV (PLHIV).

Funded by the National Institutes of Health, the International epidemiology Databases to Evaluate AIDS (IeDEA) is a global research consortium dedicated to pooling existing clinical data on PLHIV and people at risk for HIV in routine care to generate large data sets for priority research questions in HIV/AIDS treatment and care provision (Zaniewski et al., 2018). Comprised of seven regional networks IeDEA collects data from contributing sites across 44 countries, with data collection occurring from 2004 to present. The seven regional centers are the North American AIDS cohort collaboration on research and design (NA‐ACCORD) (Gange et al., 2007), the Caribbean, Central and South America Network (CCASAnet) (McGowan et al., 2007), the Asia‐Pacific, and four regions in Sub‐Saharan Africa: Central Africa, West Africa, East Africa, and Southern Africa (Chammartin et al., 2020; Egger et al., 2012). Importantly, clinics can determine their participation in the IeDEA research consortium; hence they may contribute data for some periods and not others. For this analysis, we focus on 777 clinics that have ever participated in the IeDEA research consortium from 2006 to present (Brazier et al., 2024). CCASAnet, the smallest IeDEA region, has 10 clinic locations; Southern Africa, the largest region, has 499 contributing clinic locations. Figure 4 maps both the count of clinic locations by country and IeDEA clinic region.

IeDEA data contains a wide variety of health indicators including dates of enrollment in care, antiretroviral therapy initiation, medication regimen, general visit dates, lab measurements, select biological specimens, as well as demographic variables including date of birth, sex, pregnancy status, mortality, lost‐to‐follow up, and comorbidities (IeDEA Website, 2024).

### 2.4 Analytic and Statistical Methods

We use a regression framework with a semi‐parametric generalized additive model (GAM) to assess the risk of drought and flood events for each IeDEA clinic. To assess model fit and variable selection in our specification search, we use ANOVA, AIC, and BIC as well as visual diagnostics (Gelman & Hill, 2021; Hastie et al., 2017). The dependent variables and final model specifications for each set of risks (drought and flood) are described below. Additional details of the model specification search are available in Supporting Information S1.

#### 2.4.1 Drought Exposure Risk

For drought measures, we convert the SPEI measures for each month in the 5‐month period (three wettest months and two buffer months described above) into a status of non‐drought, drought, or exceptional drought. Next, we create year‐month‐clinic‐state specific set of transition probability matrices which allows us to examine meaningful drought patterns and trends across clinic locations, regions, or years. To illustrate, consider a clinic with drought sequence: “N‐D‐E‐N‐E,” where *N* is non‐drought, *D* is drought (SPEI < −1.3), and *E* is exceptional drought (SPEI < −2). There are four total state transitions, and three that are of particular interest as they indicate a transition to a drought condition: N→D, D→E, and N→E. We can build four transition matrices (one for each month‐to‐month transition); the first of which takes the following form:

$$\begin{array}{cccc} & N & D & E\\ N & 0 & 1 & 0\\ D & 0 & 0 & 0\\ E & 0 & 0 & 0 \end{array}$$

This matrix approach is based on mathematical models of multistate transitions and regime switching, and supports many different types of analyses (Willekens, 2014). For example, we can model the probability of observing a drought in the first month of the rainy season across sites in a region (both in a given year, or year‐on‐year). Similarly, we can model the probability of observing intensifying drought (a D→E transition) in any given year.

For this analysis we focus on the probability of observing any drought condition transitions or extensions at a given clinic location, irrespective of transition month. This approach naturally eliminates the status of the first month, pre‐wet season unless a D→D,D→E,E→D, or E→E transition occurs. Specifically, we measured the following state transitions: N→D;D→E; and N→E; and drought extensions: D→D,E→E. We sum the occurrences of all five relevant transitions for each season and consider the random variable binomial distributed. In our “N‐D‐E‐N‐E” example, we observe three drought exposure occurrences of interest in four state transitions.

To rank IeDEA clinic locations by drought risk, we fit a semi‐parametric GAM of the form:

$$\text{logit}\left(\pi_{i}\right)=\alpha {\text{clinic}}_{i}+\beta X_{i}+\sum_{j}f_{j}\left({\text{year}}_{i|i\in j}\right)$$

where π=Pr(Y=1) (any drought exposure) for any given clinic (*i*); α is the clinic‐specific effect for clinics *i* = 1,2…,777; $X_{i}$ includes region‐dummy variables (*j* = 1,2,…7); β is the estimate of the region effects; and $f_{j}\left({\text{year}}_{i|i\in j}\right)$ smoothly models the non‐parametric time trends by IeDEA region to control for region‐specific changes over time with knots = 12. This parameterization is naturally conservative and will shrink coefficients toward a common mean given the region; it also assumes that the clinic‐specific effects are constant over time. We used a generalized cross‐validation (GCV) routine with zero‐sum constraint and an appropriate penalization scheme as implemented in MGCV (Hastie et al., 2017; Wood, 2011, 2017; Wood et al., 2016).

Newer scholarship suggests that typical cross‐validation techniques may be less well suited for spatially referenced data because models without a spatial cross‐validation implementation may overestimate variable importance or model accuracy (Davenport et al., 2024; Meyer & Pebesma, 2021). These constraints are especially important in the context of prediction and hypothesis‐based inference. In this paper, our focus is on recovering a relative ranking given our covariates and as such we are less concerned with precise standard errors. However, in Supporting Information S1 we conduct a sensitivity analysis of the relative ranks given upper‐ and lower‐boundaries of a 90% CI.

In this model specification, the sum of the α and β coefficients can be interpreted as *the portion of the risk of experiencing a drought exposure in any given year that can be attributed to a specific location relative to the other IeDEA network clinics*, after controlling for unobserved factors that vary by region (β) and regionally specific non‐linear trends $f_{j}\left({\text{year}}_{i|i\in j}\right)$.

#### 2.4.2 Flood Displacement Risk

For flood events we used a semi‐parametric GAM and take the number of displaced persons resulting from the event as the outcome of interest. We used the number of displaced persons rather than a binary measure of flood occurrence because flood occurrence is less regular and not strictly a seasonal occurrence like SPEI drought measures. Additionally, using the number displaced embeds a measure of flood severity in our models. Our final model to assess flood risk is as follows:

$$\log\left({\text{displaced}}_{ik}\right)=\alpha {\text{clinic}}_{i}+\beta X_{i}+\gamma \;\log\left({\text{duration}}_{ik}\right)+\psi {\text{intertices}}_{ik}+\phi \left(X_{i}\cdot Z_{i}\right)$$

where *k* indexes the unique flood event and *i* indexes the clinic. Interstices is the period between the *k*th flood and the last recorded flood for the *i*th clinic, categorized into discrete periods (unknown, less than 1 month, 1–3 months, 3–6 months, 6 months–1 year, 1–2 years, 2+ years). $X_{i}$ includes region‐dummy variables; β is the estimate of the region effects; $\phi \left(X_{i}\cdot Z_{i}\right)$ models the year‐by‐region trends linearly across all clinics. Similarly to drought, this parameterization is a conservative estimate of the deviation from a common region‐specific mean, and we interpret the linearly‐modeled sum of the α and β coefficients as the *portion of the risk of flood‐displacement exposure attributable to each clinic location relative to the IeDEA network of clinics* after controlling for space‐time specific unobserved factors.

## 3 Results

### 3.1 Climatic Zones of IeDEA Clinics

Of the 777 clinic locations, over one‐third are located in tropical, Mediterranean or semi‐arid climes (38%). Another 359 clinics are located in sub‐tropical climes, which are particularly vulnerable to changing mean temperatures and lasting water deficits (Vicente‐Serrano et al., 2010). The most common climate zone is dry‐winter subtropical, which corresponds to the high count of clinics in the Southern Africa region and particularly Zambia and South Africa (286 clinics or 36.8%). Table 1 displays the distribution of clinic locations across climatic zones.

**Table 1:** IeDEA Clinics Are Located in a Variety of Climatic Zones

Climate zone classification	Clinic count (%)
Arid	2 (0.3)
Dry‐winter subtropical	286 (36.8)
Humid continental	73 (9.4)
Mediterranean	33 (4.2)
Oceanic	103 (13.3)
Semi‐Arid	50 (6.4)
Subarctic/boreal	1 (0.1)
Tropical monsoon	21 (2.7)
Tropical rainforest	33 (4.2)
Tropical savannah	159 (20.5)

*Note*. The most common climatic zone is dry‐winter subtropical, which aligns with the concentration of clinic sites in the Southern Africa region.

### 3.2 Drought Exposure Risk

**Figure 5:**
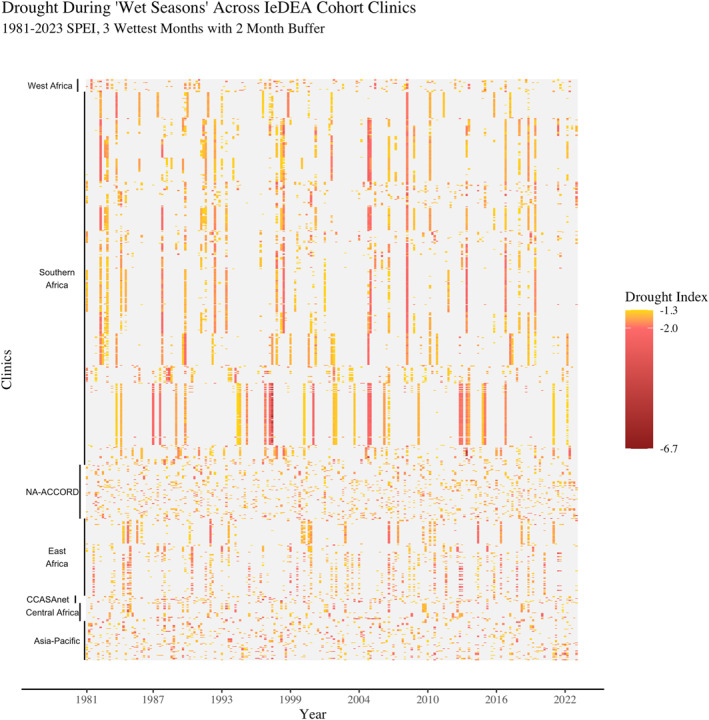
From left to right, this figure traces each IeDEA clinic location's drought history for the 1981–2023 period. Clinic locations are sorted by IeDEA region and country within‐region. Wet‐month droughts where SPEI values were less than −1.3 are highlighted in yellow; increasing severity is indicated by a transition to red. While single‐month wet‐month droughts are common to all regions, multi‐month (extended) droughts (denoted with thicker bars) seem to be most common in some areas of Southern Africa and Eastern Africa regions.

Figure 5 displays the wet‐month drought histories for all clinic locations, sorted by country and IeDEA region. Of the larger IeDEA regions, each appears to have a distinct wet‐month drought pattern. Clinics in the Southern Africa region experienced long periods of non‐drought (gray space) and then wide‐spread drought months (long vertical color bars); some stretching across multiple consecutive wet months (thicker color columns). Clinics in the NA‐ACCORD region (concentrated in the United States) had more sporadic, less widespread drought months and fewer clinics that are dispersed over a wider area. Clinics in East Africa appeared to have shorter interstices between drought events; droughts are a more regular occurrence.

**Figure 6:**
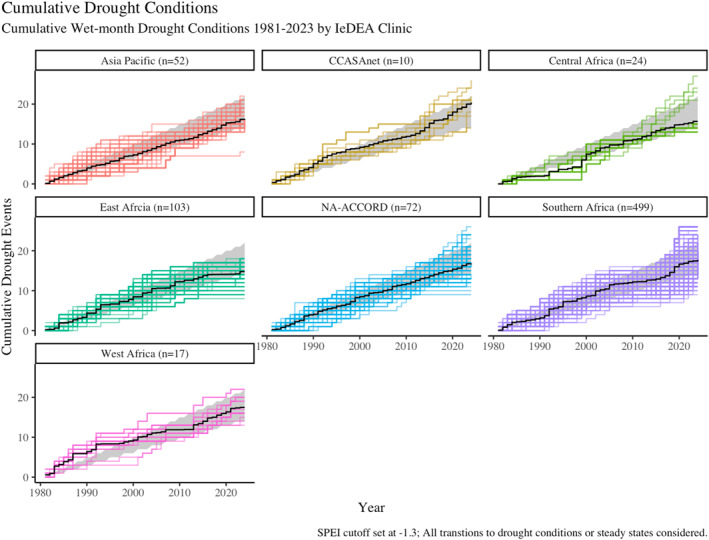
In this figure, each line indicates a single clinic location. A single step denotes one wet‐month drought condition during the corresponding year; multiple steps indicate the total number of wet‐month drought conditions observed during the corresponding year (1981–2023). The black line is the average cumulative wet‐month drought exposure for all clinics in the region. The area captured in the gray polygon indicates an every‐other‐year drought (one wet month drought) and an every‐third‐year drought (one wet month drought) for reference.

Figure 6 is a graphical representation of the drought transition matrix variable construction. It shows the cumulative wet‐month drought conditions observed in each corresponding year/season, clinic, and IeDEA region. Each colored line represents a clinic location; each step in the line indicates a wet‐season drought month occurred during that year/season (multiple vertical steps indicate more than 1 month). The black lines indicate the average cumulative wet‐month drought exposure across all clinics in a region. The gray polygon captures the area between an every‐other‐year drought (upper edge, hypothetical data) and every‐third‐year drought (lower edge, hypothetical data) as a visual sense of the frequency. There is variation between clinic exposure both within and across regions over the period. The average slope, for example, in the Asia‐Pacific region and Central Africa region tends toward the one‐in‐every‐third‐year drought edge of the gray polygon, while Southern Africa, NA‐ACCORD, and East Africa the average slope leans more toward the middle of the polygon. Different sites also experience very different cumulative drought exposures. Figure S1 in Supporting Information S1, is a non‐cumulative version of the drought transition matrix that shows the proportion of clinics in each region that experienced zero, one, two, three, or four drought transitions for each year 1981–2023.

The summed coefficients recovered from the drought model represent portion of drought risk (in the three wettest months of the year) that is specific to each clinic location. We refer to these values as clinic‐specific drought risk for ease. Values range roughly from 0 to 0.16 on the inverse logit scale ({0,1}) and the distribution has a heavy right tail. We categorized the drought risk further into low risk (lower quartile), medium risk (interquartile range) and high risk (upper quartile) to illustrate the relative rank of risk coefficients across all clinic locations. Table 2 displays the percent of clinic locations in each region in each risk category. To assess the stability of these findings we use the same ranking technique but evaluate each clinic coefficient at the upper and lower 90% confidence bounds (included in the sensitivity analysis in Supporting Information S1). The results and discussion in Supporting Information S1 support the methods presented here. In general, participating clinics in NA‐ACCORD are at a comparatively high and medium clinic‐specific drought risk relative to other clinic locations, similarly with the Southern Africa region. Surprisingly, no East Africa clinic locations have high clinic‐specific drought risk values as compared to other IeDEA clinics. Similarly surprising, relatively many clinics from the Asia‐Pacific region do have high risk values compared to other IeDEA clinics and regions. (Table S1 in Supporting Information S1 shows the region fixed‐effect estimates (regional means) over time).

**Table 2:** This Table Shows the Percent Contribution of Each IeDEA Region to the Risk Categories for Drought and Flood‐Displacement Events

Drought risk in percent of all clinics (*n*)			
	Low risk (p<0.45)	Medium risk (0.45≥p≤0.68)	High risk (p>0.68)
Asia‐Pacific	0.1 (1)	4.5 (35)	2.1 (16)
CCASAnet	0 (0)	0.3 (2)	1 (8)
Central Africa	0.9 (7)	1.7 (13)	0.5 (4)
East Africa	5.3 (41)	8.0 (62)	0 (0)
NA‐ACCORD	1 (8)	4.5 (35)	3.7 (29)
Southern Africa	17.2 (134)	30 (233)	17 (132)
West Africa	0.3 (2)	1.3 (10)	0.6 (5)

Flood‐displacement risk in percent of all clinics (*n*)			
	Low risk (p<0.43)	Medium risk (0.43≥p≤0.61)	High risk (p>0.61)
Asia‐Pacific	1.0 (7)	1.4 (10)	4.7 (34)
CCASAnet	0.1 (1)	0.4 (3)	0.8 (6)
Central Africa	0 (0)	2.4 (17)	0.4 (3)
East Africa	4.3 (31)	1.4 (10)	8.6 (62)
NA‐ACCORD	6.0 (43)	1.5 (11)	1.2 (9)
Southern Africa	4.6 (33)	38.4 (277)	20.4 (147)
West Africa	0.1 (1)	0.3 (2)	1.9 (14)

*Note*. These results are reproduced and relatively stable in the sensitivity analysis, which incorporates 90% confidence bounds. Note that 56 clinics had no history of flood exposure.

**Figure 7:**
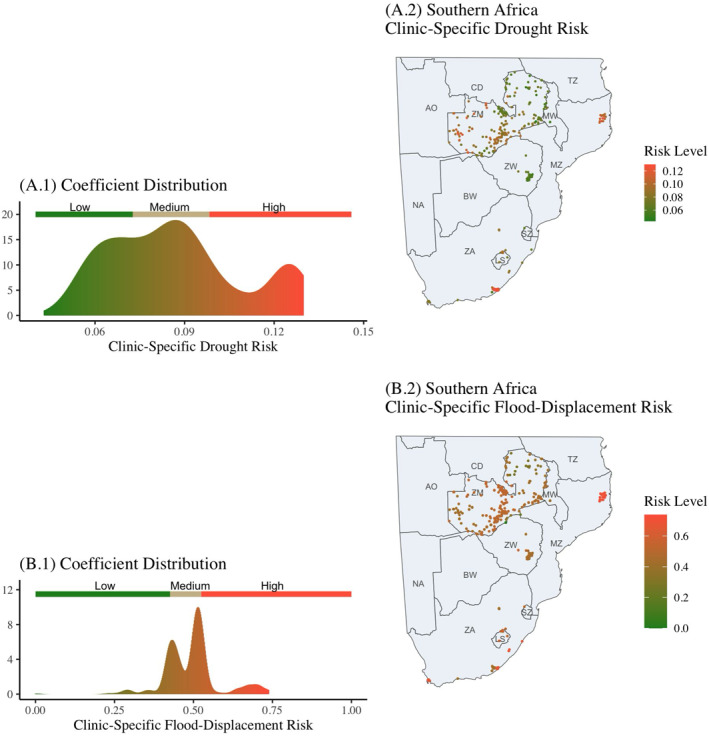
The clinic‐specific coefficient estimates from the drought model are displayed for the Southern Africa IeDEA region in Panel (a). Panel (a1) graphically displays the density of summed coefficients for each clinic location. The low, medium, high colored bar denotes the lower quartile, interquartile range, and upper quartile from the entire (global) distribution of the summed clinic coefficients. Panel (a2) at the right displays the clinic locations and coefficient value (clinic‐specific drought risk) spatially. Clinic‐specific drought risk is clustered similarly to point clusters, as expected, and intracountry variation is also apparent. In Panel B, the summed clinic‐specific flood coefficient estimates from the model are displayed for the Southern Africa IeDEA region. Panel (b1) graphically displays the density of coefficients for each clinic location. The low, medium, high colored bar denotes the lower quartile, interquartile range, and upper quartile from the entire (global) distribution of the summed flood coefficients. Panel (b2) at the right displays the clinic locations and coefficient value (clinic‐specific flood‐displacement risk) spatially. Flood‐displacement risk has a less pronounced spatial pattern from the drought coefficients (Note coefficients have been min‐max scaled for easy interpretation on {0,1}).

Focusing on the Southern Africa IeDEA region, the left panel of Figure 7a1 shows the distribution of clinic‐specific drought risk (from the summed model coefficients) for all clinics in the region compared to the overall risk categories calculated shown in the colored bar above the distribution (calculated in Table 2). Figure 7a2 maps the clinic‐specific drought risk to the clinic locations. The clinic‐specific drought risk level is clustered similarly within point clusters, as expected, and there are also areas of high risk such as in Northeast Mozambique, coastal South Africa and western Zambia.

### 3.3 Flood Displacement Risk

**Figure 8:**
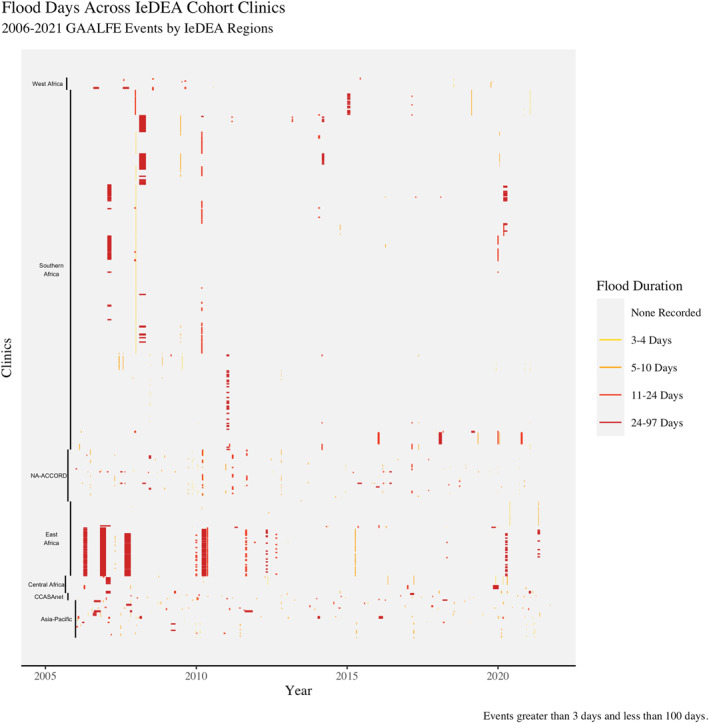
From left to right, this figure traces each IeDEA clinic location's flood exposure history for the 2006–2022 period. Clinic locations are sorted by region and country‐in‐region, and flood exposures are colored by the duration of the flood event.

As Figure 8 shows, flood events are much less common than drought events over the period (2006–2022). Flood events are generally more frequent in the Asia‐Pacific IeDEA region and the NA‐ACCORD region. Longer duration flood events are more common in the East Africa IeDEA region.

**Figure 9:**
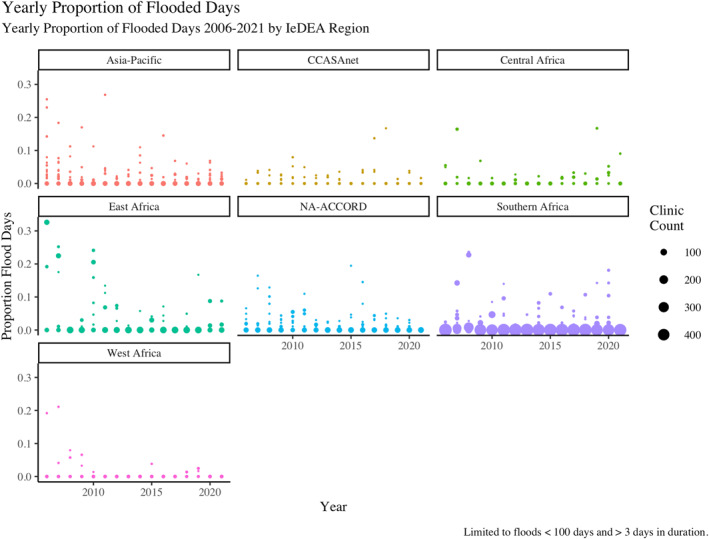
In this figure, points are sized to represent the number of clinics with the same proportion of exposed days in the same year. Generally the proportion of clinics with 20% or more exposed days is small across all regions.

The proportion of days per year of flood exposure is presented graphically in Figure 9. Generally, most clinics experience less than 10% of flood exposure days for any given year, though there is some variation apparent by both year and region. The East Africa region has the highest average proportion of flooded days from 2006 to 2022, with an average of 3.3% per clinic.

Similarly to the drought coefficients, we recover the summed clinic and region coefficients as the clinic‐specific effect on flood‐driven displacement from the flood displacement models. For ease of interpretation, we use a min‐max transformation to cast values to a {0,1} range to match the clinic‐specific drought risk. (Note that 56 clinics had no flood exposure/displacement during the 2006–2022 period and were excluded from the analysis.) We refer to the clinic‐specific effect on flood‐driven displacement as the clinic‐specific flood‐displacement risks. The clinic‐specific flood‐displacement risk coefficients are roughly symmetrically distributed around 0.5, but the distribution is kurtotic. We categorized the clinic‐specific flood‐displacement risk coefficients into low risk (lower quartile), medium risk (interquartile range) and high risk (upper quartile) to illustrate the relative rank of clinic‐specific flood‐displacement risk coefficients across all clinic locations. The lower half of Table 2 shows the percent contribution of each IeDEA region for each risk category.

Focusing on the Southern Africa IeDEA region, Figure 7b1 shows the distribution of clinic‐specific flood‐displacement risk for all clinics in the region compared to the overall risk categories (from Table 2) represented in the colored bar. Figure 7b2 maps the clinic‐specific flood‐displacement risk to the clinic locations spatially for the Southern Africa IeDEA region. Flood‐displacement risk is somewhat clustered, but has a much less definitive spatial patterning than drought.

Comparing Figures 7a2 and 7b2 there appears to be areas at high risk of both flood and drought. The area in the northwest of Mozambique, for example, has a clear multi‐hazard profile. Table 3 shows the counts of IeDEA clinics for both flood‐displacement and drought (multi‐hazard risk) as well as multi‐hazard risk by IeDEA region. The results of a $\chi^{2}$ test for independence on the dual risk classification section of Table 3 suggest that flood and drought exposures are not independent (*p* < 0.05). Globally, 5.9% of IeDEA clinics (*n* = 46) are at a high multi‐hazard risk. These clinic locations are globally dispersed, with five of seven regions having at least one clinic location at high multi‐hazard risk. The countries of Mozambique and South Africa have the highest counts of high‐risk multi‐hazard clinics, though in Mozambique these clinics are very spatially concentrated.

**Table 3:** Multi‐Hazard Risk Categories Are Based on Flood and Drought Risk Quartiles Combinations

Multi‐hazard risk			
	Flood classification		
Drought classification	Low risk	Medium risk	High risk
Low Risk	32	127	32
Medium Risk	62	228	97
High Risk	22	75	46

Multi‐hazard risk by region			
Region	Low risk	Medium (mixed) risk	High risk
Asia‐Pacific	0	49	2
CCASAnet	0	5	5
Central Africa	0	20	0
East Africa	7	96	0
NA‐ACCORD	5	50	8
Southern Africa	20	408	29
West Africa	0	15	2

*Note*. High risk clinics are those in the upper quartile of the flood and drought coefficients. Low risk clinics are those in the lower quartile of the flood and drought coefficients. The “Medium (Mixed) Risk” category contains clinic locations with all other combinations of risk categories; the 56 clinic locations with no observed flood displacement are excluded from this table.

## 4 Discussion

In this paper, we have described a climate‐science and GIS‐based procedure to identify both drought and flood exposure risk from 1981 to 2023 and 2006 to 2021, respectively, for clinics within the IeDEA cohort, using publicly available weather data. We identified drought months that occur during a typical wet‐season for all clinic sites across the globe, which are particularly dangerous for subsistence farmers, agriculturalists, and pastoralists as they can be linked to crop failures and livestock loss (Funk et al., 2019; Rosegrant & Cline, 2003). To determine flood exposure and related displacements, we use a news and satellite‐based reporting system for global flood events (GAALFE) and determine exposure based on location of a clinic site within a flood event. Our results suggest substantial variation both within and between IeDEA regions to both flood and drought events over the period, though floods were less common overall. Where wet‐month droughts are frequent among the clinics, extended (multi‐month) wet‐month droughts were most visible in the Southern Africa and Eastern Africa regions. The frequency, variation, and potential severity of exposure to extreme drought underscores the importance of understanding its impact on care availability and outcomes. For flood, though less frequent, there is likely variation in severity and infrastructure damage that are worthy of further investigation. In sum, the frequency and variation we observe in both flood and drought are likely adequate to support future comparative exposure research designs.

Additionally, we also demonstrate a statistical approach to identify clinics with a high‐risk for flood/drought relative to other network clinics and identify clinic locations where risk for both flood and drought is relatively high (high multi‐hazard). The clinics identified in the upper quartile may be of interest for future research designs. On the one hand, because of the way our models are constructed, these clinics reflect higher relative exposure levels to drought/flood over the last several decades, and as a result, both clinics and their patient populations may experience more frequent negative outcomes (such as care disruption). On the other hand, because of their high relative exposure levels, these clinics and patient populations may also be more adapted to drought/flood exposure than other areas and therefore may not experience more frequent negative outcomes. While this paper does not examine these effects directly, it does provide a historic record of drought and flood exposure that will be leveraged in future place‐specific research designs. For example, researchers could leverage this historical record to examine flood/drought naive exposures or depletion of adaptive resources after a multi‐year drought exposure.

Our analysis has several limitations. First and foremost is the generalizability of our findings. Southern Africa largely drove our overall estimates of drought and flood‐displacement exposure risk due to the sheer number of clinics in our sample with a high risk of flood, with a high risk of drought, and with a high multi‐hazard risk. We emphasize that the models and findings described in this paper are not suitable for spatial‐out‐of‐sample prediction (i.e., new clinic locations), and we do not use our standard errors for hypothesis testing. Instead, our results are aimed at application; per our methods and definitions, clinic‐specific exposures are absolutes (exposed/unexposed/exposure intensity), and our modeling approach ranks exposure probability relative to other IeDEA network clinics over a multi‐year period. These measures could highlight potential areas of focus for future causal designs. We also restrain our drought measure to the monthly scale. While 3‐ or 5‐month SPEI scales may be more stable and offer additional insights, there is evidence to suggest that shorter‐term droughts have pronounced effects on growing seasons (Pendergrass et al., 2020; Zeng et al., 2023). Future analyses, depending on research questions, could modify our methodology to accommodate longer (or shorter) time scales. By limiting our drought exposure to wet‐month droughts, we necessarily miss droughts that occur during other seasons/months; while there could be downstream effects of these droughts on human health (e.g., heat exposure, dehydration), our focus on wet‐month droughts is also aligned with crop failures/uncertainty. Another important limitation is embedded in our geographical treatment of the clinic locations. There is some uncertainty in both clinic location (coordinates) and clinic catchment area. IeDEA does not capture patient residential location, and it is likely that the catchment areas of different IeDEA‐participating clinics vary in size in a systematic way globally (consider the catchment area for an urban clinic in the United States vs. a clinic in rural sub‐Saharan Africa). In this paper, we essentially assume that patients and clinics share the same exposures; this assumption may be more valid in some locations/research designs than others.

A final limitation comes from our environmental data inputs for drought and flood. Again, there is inherent uncertainty in the gridded data products used in our exposure cutoffs and statistical models, and the accuracy of these measures may be spatially biased. (For interested readers, there is a literature on the benefits of integrating station data that obliquely addresses this; see Beguería et al., 2014; Funk et al., 2015; Vicente‐Serrano et al., 2010 to start.) This is the nature of gridded data products and one of the strengths of integrating station data when available, as CHIRPS does. Similarly, flood events are harder to detect globally. GAALFE sources its flood events from news, governmental, instrumental, and remote sensing sources, and certainly (historically) contains bias from each source. However, GAALFE continues to improve as instrumentation costs decrease, news sources become more representative and satellite imagery becomes more available. In our analysis, we only generate flood exposures from 2006 forward to avoid earlier, more biased, flood information.

Despite these limitations, our analysis has many strengths. It combines foundational elements of climate science (e.g., SPEI, global climatology) with statistical methods found in the social sciences (e.g., state transition theory) to create a record of historical drought and flood event exposures for a consortium of HIV care clinics across the globe. By considering only wet‐month droughts in our methodology, we highlight the link between rainy/growing seasons, drought conditions, and potential crop loss. By considering the number of displaced individuals for each flood event, we reflect a measure of severity in our flood risk model. Finally, this paper contributes a sound reproducible methodology to determine drought and flood exposures and identify higher risk clinic locations for future work to examine the effects of EWEs on HIV care and care outcomes, perhaps even in a projection framework with different climate scenarios. While this application is specific to the clinics included in our analysis, the methodology is generalizable to other networks of care and is an important step in assessing impact of climate change on health care service provision and related health outcomes.

Ideally, a goal of future research should also be to identify causal mechanisms between EWE exposure and health outcomes for PLHIV. However, identifying causal mechanisms is difficult methodologically. In a causal inference framework, there is an important trade‐off between geographic variation and precision of the causal identification strategy, which may impact the generalizability of findings. For example, consider a hypothetical 2‐year randomized controlled trial design aimed to assess the causal effects of EWE exposures on health outcomes. Such a trial aimed at a geographically clustered set of clinics or villages may provide a precise estimate of an EWE exposure effect (a treatment such as drought, flood or multi‐hazard), but the EWE exposure itself is likely limited both spatially and temporally in such a design (such as low heterogeneity of exposure due to spatial clustering). On the other end of the spectrum, consider a repeated cross‐sectional design. Such a sample spread over a large spatial extent may provide good variation in EWE exposures (and potential treatment), but the causal identification strategy may rely on overly strong assumptions (such as assumed homogeneity of exposure across a dispersed landscape). Longitudinal multi‐site cohort study designs, including quasi‐experimental methods that leverage some of the randomness inherent in EWEs, are a potentially fruitful middle ground. With a greater potential for variation in exposures or treatments as well as careful attention to exposure measurement, selection bias, competing or linking pathways, and identification strategies, such designs could yield more robust internally and externally valid findings.

## 5 Conclusion

In this paper, we have developed procedures for characterizing the occurrence, intensity, and duration of drought, flood and multi‐hazard exposures. When applied to an international network of HIV care clinics, we found the burden of such exposures to be quite high and variable enough to support additional (potentially causal) research designs aimed at characterizing the impacts of EWEs on health outcomes. The results and methodology in this paper opens the door for investigation of new and important questions for care providers, funding agencies, and policymakers with an HIV care delivery focus for the 39.9 million people living with HIV and 650,000 people with new HIV infections annually who require ongoing access to healthcare and medication for the remainder of their lives. If the frequency and duration of drought and flood events increases with climate change, how will care provision be affected? Does an HIV clinic's exposure to drought during a wet season cause food insecurity and lower medication adherence during the following harvest season? Where should resources intended to bolster climate change resilience for PLHIV be targeted? This paper provides the methodology to determine drought and flood exposures in the first step toward answering such questions.

## Conflict of Interest

The authors declare no conflicts of interest relevant to this study.

## Supporting information

Supporting Information S1

## Data Availability

IeDEA clinic locations (approximate coordinates) are available to researchers with appropriate credentials (including Internal Review/Human Subjects training). Data for this research are not publicly available due to pre‐existing human subject agreements and protection of private health information (https://www.iedea.org/). Clinic coordinate data are stored in this in‐text citation for reference Brazier et al. (2024). Precipitation data sets are publicly accessible from the Climate Hazards Center (CHC) CHIRPS v2 data (https://www.chc.ucsb.edu/data/chirps) (Funk et al., 2015) and merged with a Global Reference Evapotranspiration data set from Physical Science Laboratory (NOAA PSL) (https://www.psl.noaa.gov/eddi/globalrefet) (Hobbins et al., 2016; McEvoy et al., 2016). Monthly SPEI was derived using R package SPEI version 1.8.1 for each averaged 100 km area around each clinic location (100 km radius) (Beguería & Vicente‐Serrano, 2023). Wettest months were determined using CHPclim 2.0 available from the CHC (https://data.chc.ucsb.edu/products/CHPclim/) (Funk et al., 2015). Flood data are publicly available from GAALFE (https://floodobservatory.colorado.edu/) with supporting documentation. Precipitation during the flood events comes from the CHC's CHIRPS v2 data set, with summed values for all grid cells within 25 km of a clinic location during the flood period (25 km radius). All figures and maps were made using R version 4.5.0, with “ggplot2” version 3.5.1, “sf” version 1.0‐21, base graphics and “patchwork” version 1.3.0, freely available via the CRAN network (https://CRAN.R‐project.org).
