# Supplementary material for: Modeling the Burden of Extreme Weather Events in a Large Network of International HIV Care Cohorts
Source: Geohealth. 2025 Nov 3;9(11):e2025GH001514. doi: 10.1029/2025GH001514 (PMC12583239; doi:10.1029/2025GH001514)
Supplement: Supplementary file 1 — Supporting Information S1 [file GH2-9-e2025GH001514-s001.docx]

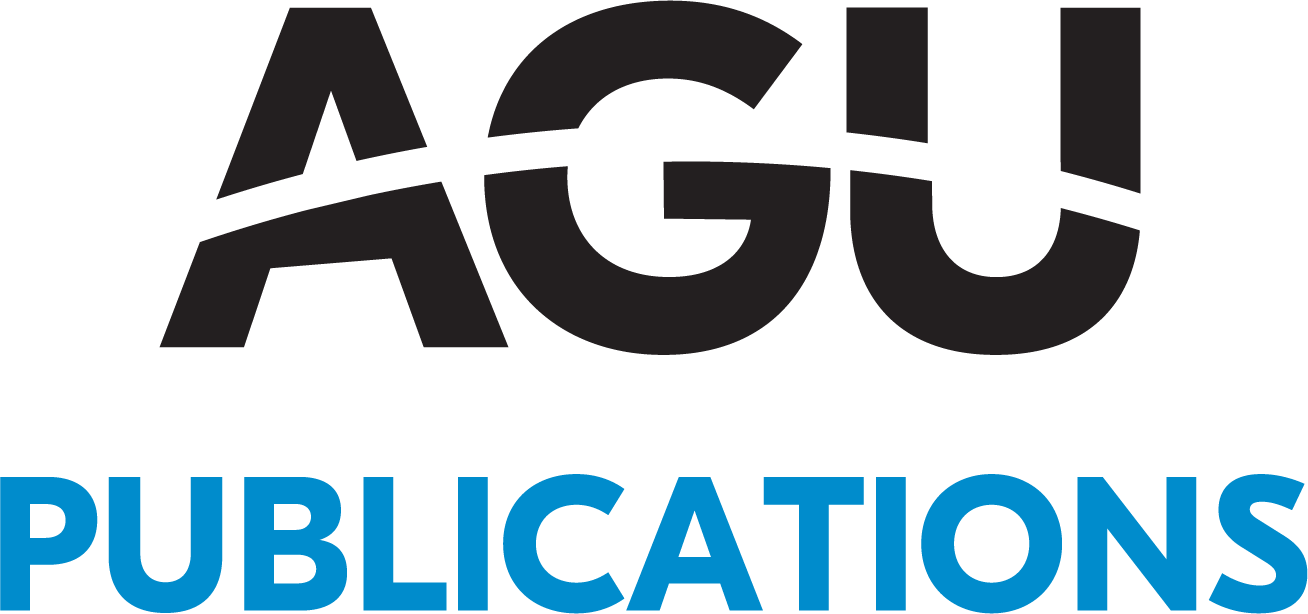


*GeoHealth*

Supporting Information for

**Modeling the Burden of Extreme Weather Events in a Large Network of International HIV Care Cohorts**

Sophia D. Arabadjis^1,2^, Frank Davenport^3^, Ana Maria Vecedo Cabrera^4,5^, Zachary Shahn^1^, Ellen Brazier^1^, Andrew Maroko^1,6^, Avantika Srivastava^1,7^, Gad Murenzi^8^, Timothy John Dizon^9^, Keri N. Althoff^10^, Antoine Jaquet^11^, Aggrey S. Semeere^12^, Yanink Caro Vega^13^, Mark K. U. Pasayan^9^, Sheri D. Weiser^14^, Denis Nash^1,7^

^1^Institute for Implementation Science in Population Health, City University of New York, Graduate School of Public Health and Health Policy, New York, NY, United States

^2^University of California Center for Climate Health Equity, University of California San Francisco, San Francisco, CA

^3^Climate Hazards Center, Department of Geography, University of California, Santa Barbara, CA

^4^Institute of Social and Preventive Medicine, University of Bern, Bern, Switzerland

^5^Oeschger Center for Climate Change Research, University of Bern, Bern, Switzerland

^6^Institute for Health Equity Research, Icahn School of Medicine at Mount Sinai, New York City, NY

^7^Department of Epidemiology and Biostatistics, City University of New York, New York City, NY

^8^Research for Development and Rwanda Military Referral and Teaching Hospital, Kigali, Rwanda

^9^Research Institute for Tropical Medicine, Department of Health, Republic of the Philippines

^10^Department of Epidemiology, Johns Hopkins Bloomberg School of Public Health, Baltimore, MD

^11^National Institute for Health and Medical Research (INSERM) UMR 1219, Research Institute for Sustainable Development (IRD) EMR 271, Bordeaux Population Health Centre, University of Bordeaux, Bordeaux, France.

^12^Infectious Diseases Institute, Makerere University, Kampala, Uganda.

^13^Departamento de Infectología, Instituto Nacional de Ciencias Médicas y Nutrición Salvador Zubirán, Mexico City, Mexico

^14^Division of HIV/AIDS, San Francisco General Hospital, University of California at San Francisco, San Francisco, CA

**Contents of this file**

Figure S1 Yearly Wet-month Drought Transitions by Region

Table S1 IeDEA Region Fixed Effects

Table S2 Drought Model Specification Details

Table S3 Flood Model Specification Details

Text S1 Sensitivity Analysis

Table S4 Clinic Stability in Drought Risk Categories: Upper-Bound Values

Table S5 Clinic Stability in Drought Risk Categories: Lower-Bound Values

Figure S2 Drought Comparison in Southern Africa and Asia Pacific

Table S5 Clinic Stability in Flood Risk Categories: Upper-Bound Values

Table S6 Clinic Stability in Flood Risk Categories: Lower-Bound Values

**Introduction**

The supplementary information provided here consists of three parts: additional visualizations, additional results and details of model specifications, and a sensitivity analysis of main results.

**
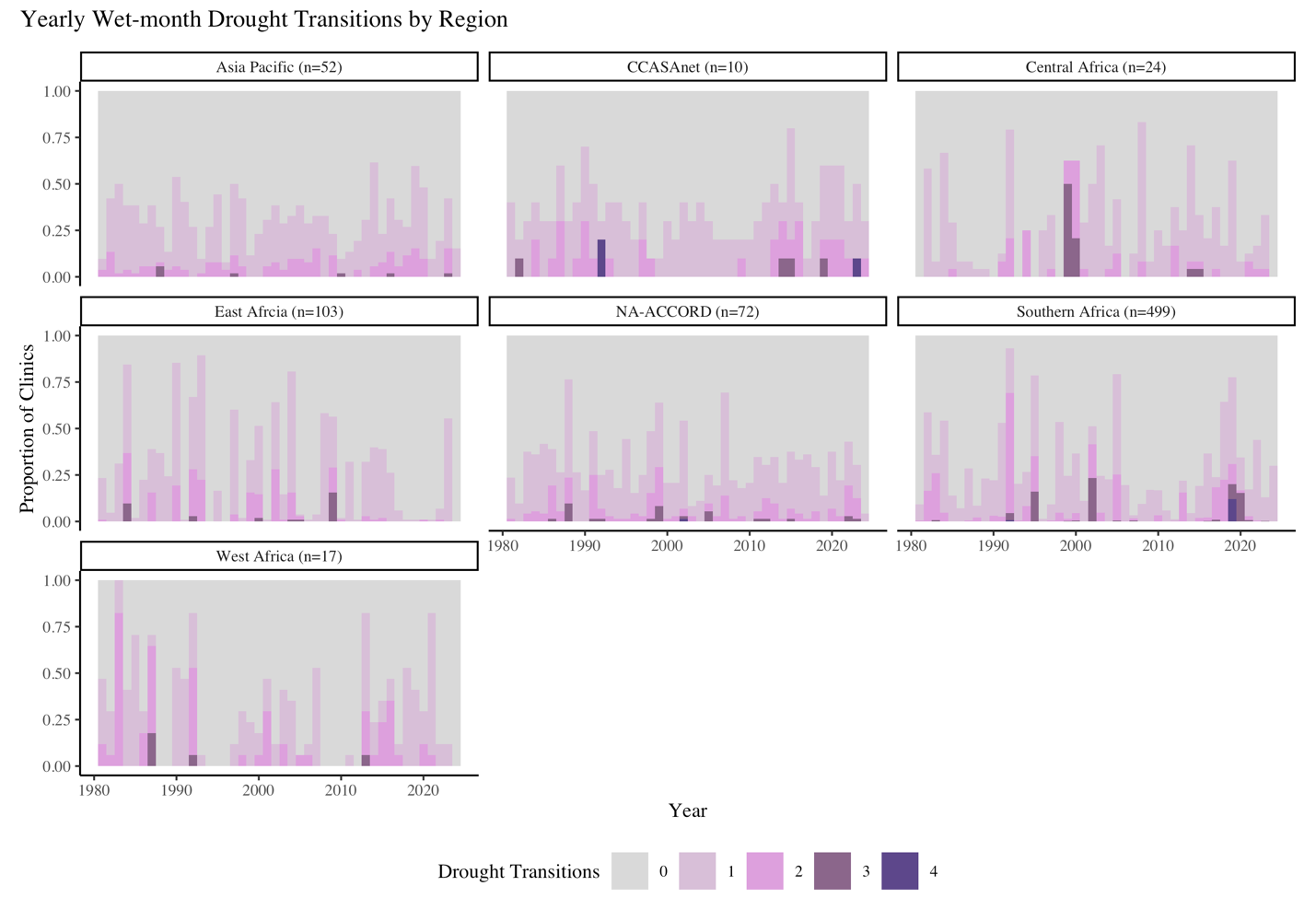
**

***Figure S1*** *This graphic displays the proportion of clinics affected by drought transitions in each region for the 1981-2023 period. The proportion of clinics in a region that had experienced no drought transitions (0 wet-month droughts) are shaded in grey, increasing drought exposures (1 month, 2 month, 3 month and all 4 months) are displayed in shades of purple. Only the regions of Southern Africa and CCASAnet had any clinics that experienced 4 drought transitions (a drought for every wet-month).*

| Region | Flood | Drought  $(e^{\theta}/(1+e^{\theta}))$ |
| --- | --- | --- |
| Asia-Pacific | 6.86 | -2.29 (0.092) |
| CCASAnet | 7.12 | -2.09 (0.111) |
| Central Africa | 5.30 | -2.44 (0.080) |
| East Africa | 7.21 | -2.53 (0.074) |
| NA-ACCORD | 3.39 | -2.29 (0.092) |
| Southern Africa | 5.73 | -2.4 (0.083) |
| West Africa | 5.17 | -2.35 (0.087) |

***Table S1*** *IeDEA region fixed effects estimates from flood and drought models.*

| Drought Model | AIC | BIC | Covariates |
| --- | --- | --- | --- |
| Final Model | 55365.84 | 62313.50 | clinic id, region, spline (year by region) |
| Alternate Specification 2 | 58335.05 | 64951.72 | clinic id, linear (year by region) |
| Alternate Specification 3 | 58447.62 | 65013.65 | clinic id, spline (year) |
| Alternate Specification 1 | 58493.80 | 65051.40 | clinic id |

***Table S2*** *AIC and BIC for the final drought model as compared to alternate specifications specifically with regards to time and region trends for the drought models. Given how we constructed our outcome variable (via transitions), there are limited covariates with which to draw on; these specification search mostly focused on the time-region trends. Note that BIC tends to select more parsimonious models, and AIC tends to select more complex models. The final model chosen minimized the BIC and AIC and had better graphical diagnostics (not shown.)*

| Flood Model | AIC | BIC | Covariates |
| --- | --- | --- | --- |
| Final Model | 11096.73 | 15804.36 | clinic id, linear (region by year), log(duration), inter-flood spacing |
| Alternate Specification 5 | 12396.85 | 16646.06 | clinic id, region, duration, inter-flood spacing, spline (year by region) |
| Alternate Specification 4 | 12398.40 | 16653.36 | clinic id, accumulated rainfall, inter-flood spacing, spline (year by region) |
| Alternate Specification 6 | 12509.87 | 16759.41 | clinic id, accumulated rainfall, spline (year by region) |
| Alternate Specification 3 | 12588.55 | 16819.10 | clinic id, accumulated rainfall, inter-flood spacing, linear (region by year) |
| Alternate Specification 1 | 12685.72 | 16881.78 | clinic id, accumulated rainfall, inter-flood spacing, region, spline (year) |
| Alternate Specification 2 | 12753.40 | 16943.71 | clinic id, accumulated rainfall, inter-flood spacing, region |

***Table S3*** *AIC and BIC for the final flood model as compared to alternate specifications specifically with regards covariate selection and time-region trend specification. In the flood models, more covariates were available to try to model displaced persons including accumulated rainfall during the flood period, inter-flood period (interstices), duration of flooded period, and trends by year and region. Note that BIC tends to select more parsimonious models, and AIC tends to select more complex models. The final model chosen minimized the BIC and AIC and had better graphical diagnostics (not shown.)*

**Text S1. Sensitivity Analysis**

The sum of $\alpha$ and $\beta$coefficients from our model specifications indicate the portion of risk of experiencing a drought or flood in any given year that can be attributed to a specific location relative to the other IeDEA network clinics -- specifically after controlling for unobserved region factors and region-specific time trends (linear or otherwise). Effectively, we are interested in how these coefficients *rank* within and across IeDEA regions. The quantile categories of low risk ($\leq{25}^{th}$percentile), medium risk (IQR) and high risk ($>{75}^{th}$ percentile) are an easy entry point into quantifying these rankings across regions.

To assess the stability of these rankings within the assumptions of the model estimates, we make use of a 90% confidence interval construction. For the drought model, the upper-bound of the confidence interval would be:

$$\mathrm{logi}t^{-1}\left( \mathrm{es}t_{u} \right)=\hat{\alpha}+t_{0.95,df} \times se_{\hat{\alpha}}+\hat{\beta}+t_{0.95,df} \times se_{\hat{\beta}}$$

and similarly for the lower edge of the confidence interval:

$$\mathrm{logi}t^{-1}\left( \mathrm{es}t_{l} \right)=\hat{\alpha}+t_{0.05,df} \times se_{\hat{\alpha}}+\hat{\beta}+t_{0.05,df} \times se_{\hat{\beta}}$$

In this sensitivity analysis we calculate the upper and lower values of the coefficients of interest, and re-rank them according to the same present quantile categories. The share of clinics that are present in the same quantile category as the original estimate are considered stable across the confidence interval. The share of these clinics are presented as % in the tables below, where the numerator is the number of observed clinics in the quantile category from the upper or lower bounds of the confidence interval, and the denominator is the number of observed clinics in the quantile category per the coefficient estimates themselves. The furthest right column indicates the number of clinics that effectively changed categories at the upper- (or lower-) bound values.

| **Clinic Stability in Drought Risk Categories: Upper-Bound Values** | | | | |
| --- | --- | --- | --- | --- |
| Region | Low Risk  %, ${n/n}_{total}$ | Medium Risk  %, ${n/n}_{total}$ | High Risk  %, ${n/n}_{total}$ | Changed Categories  $n$ |
| Asia-Pacific | 100%, 1/1 | 0%, 0/35 | 0%, 0/16 | 51 |
| CCASAnet | NA | 0%, 0/2 | 75%, 6/8 | 4 |
| Central Africa | 100%, 7/7 | 46%, 6/13 | 100%, 4/4 | 7 |
| East Africa | 54%, 22/41 | 100%, 62/62 | NA | 19 |
| NA-ACCORD | 100%, 8/8 | 100%, 35/35 | 100%, 29/29 | 0 |
| Southern Africa | 70%, 94/134 | 91%, 212/233 | 100%, 132/132 | 61 |
| West Africa | 100%, 2/2 | 100%, 10/10 | 40%, 2/5 | 3 |

***Table S4*** *This table describes the stability of each clinic in each drought risk category given the ranking variable is calculated at the upper bound of the 90% confidence interval from the final models. In each column the % is the number of clinics in the risk category from the upper bound ranking divided by the number of clinics in the risk category from the mean estimate ranking; fractions given following. A value of 100% indicates the same exact clinics appeared in that category both at the mean estimate and upper bound. A value of NA means no clinics were observed in that category based on the estimate mean. The last column indicates how many clinics changed categories when the ranking variable is calculated at the upper bound of the 90% confidence interval.*

| **Clinic Stability in Drought Risk Categories: Lower-Bound Values** | | | | |
| --- | --- | --- | --- | --- |
| Region | Low Risk  %, ${n/n}_{total}$ | Medium Risk  %, ${n/n}_{total}$ | High Risk  %, ${n/n}_{total}$ | Changed Categories  $n$ |
| Asia-Pacific | 0%, 0/1 | 0%, 0/35 | 100%, 16/16 | 36 |
| CCASAnet | NA | 50%, 1/2 | 100%, 8/8 | 1 |
| Central Africa | 0%, 0/7 | 100%, 13/13 | 100%, 4/4 | 7 |
| East Africa | 98%, 40/41 | 100%, 62/62 | NA | 1 |
| NA-ACCORD | 88%, 7/8 | 100%, 35/35 | 76%, 22/29 | 8 |
| Southern Africa | 99%, 133/134 | 94%, 219/233 | 76%, 100/132 | 47 |
| West Africa | 0%, 0/2 | 100%, 10/10 | 100%, 5/5 | 2 |

***Table S5*** *This table describes the stability of each clinic in each drought risk category given the ranking variable is calculated at the lower bound of the 90% confidence interval from the final models. In each column the % is the number of clinics in the risk category from the lower bound ranking divided by the number of clinics in the risk category from the mean estimate ranking; fractions given following. A value of 100% indicates the same exact clinics appeared in that category both at the mean estimate and lower bound. A value of NA means no clinics were observed in that category based on the estimate mean. The last column indicates how many clinics changed categories when the ranking variable is calculated at the lower bound of the 90% confidence interval.*

**
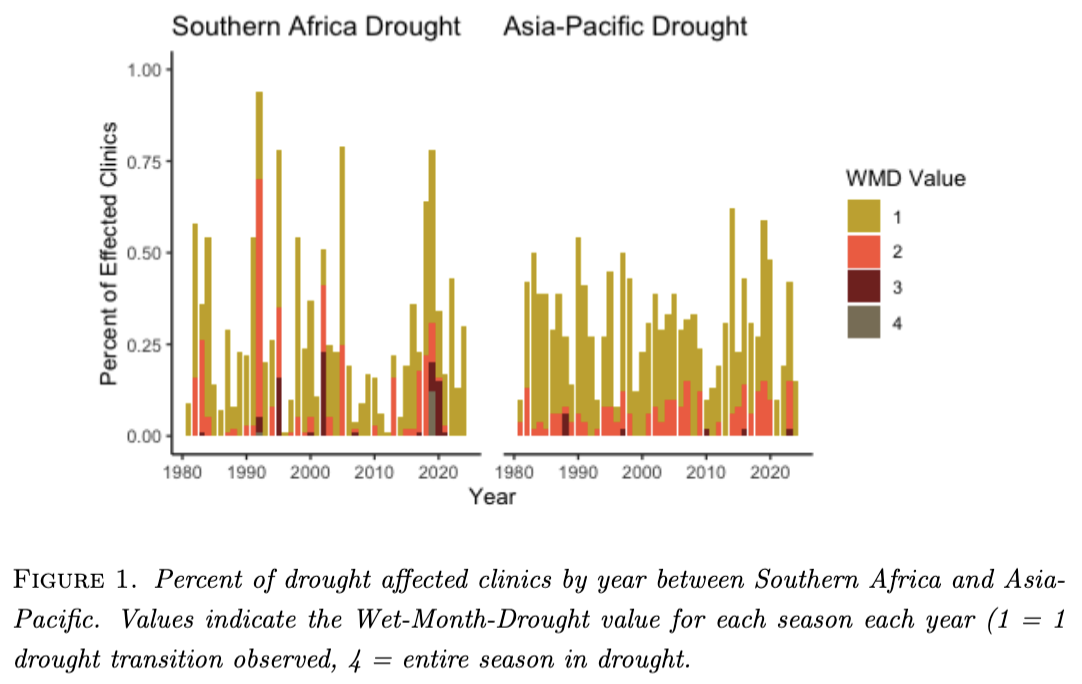
**

***Figure S2*** *Percent of drought affected clinics by year between Southern Africa and Asia-Pacific. Values indicate the Wet-Month-Drought value for each season each year (1 = 1 drought transition observed, 4 = entire season in drought.*

The drought tables indicate relative stability, meaning relatively few clinics moved risk categories when risk was based on the upper- or lower-bound of the 90% confidence intervals for each clinic (as opposed to the mean). The notable exception to this is the Asia-Pacific region which did experience larger shifts across categories depending on where in the confidence interval the quantiles were applied. At the upper-bound, the remaining Asia-Pacific sites moved to the low-risk group; at the lower-bound 36 sites moved to the high-risk group. The Asia-Pacific region has relatively fewer clinics (52) and they are less clustered than clinics in sub-Saharan Africa. They also experience less variability in the percent of affected clinics year-to-year in terms of drought (see Figure 1), which from a modeling perspective would make the estimates more stable *relative to other clinics*. Because the focus is rankings, this shift is potentially indicative of more variability in other regions as opposed to Asia-Pacific specifically. These findings, while complex spatially, give us confidence in the results presented in the paper.

| **Clinic Stability in Flood Risk Categories: Upper-Bound Values** | | | | |
| --- | --- | --- | --- | --- |
| Region | Low Risk  %, ${n/n}_{total}$ | Medium Risk  %, ${n/n}_{total}$ | High Risk  %, ${n/n}_{total}$ | Changed Categories  $n$ |
| Asia-Pacific | 92%, 11/12 | 0%, 0/9 | 47%, 14/30 | 26 |
| CCASAnet | 0%, 0/1 | 67%, 2/3 | 33%, 2/6 | 6 |
| Central Africa | NA | 100%, 17/17 | 0%, 0/3 | 3 |
| East Africa | 6%, 2/31 | 36%, 5/14 | 10%, 6/58 | 90 |
| NA-ACCORD | 51%, 26/51 | 33%, 1/3 | 22%, 2/9 | 34 |
| Southern Africa | 39%, 33/85 | 53%, 164/312 | 27%, 16/60 | 244 |
| West Africa | 0%, 0/1 | 100%, 2/2 | 100%, 14/14 | 1 |

***Table S6*** *This table describes the stability of each clinic in each flood risk category given the ranking variable is calculated at the upper bound of the 90% confidence interval from the final models. In each column the % is the number of clinics in the risk category from the upper bound ranking divided by the number of clinics in the risk category from the mean estimate ranking; fractions given following. A value of 100% indicates the same exact clinics appeared in that category both at the mean estimate and upper bound. A value of NA means no clinics were observed in that category based on the estimate mean. The last column indicates how many clinics changed categories when the ranking variable is calculated at the upper bound of the 90% confidence interval.*

| **Clinic Stability in Flood Risk Categories: Lower-Bound Values** | | | | |
| --- | --- | --- | --- | --- |
| Region | Low Risk  %, ${n/n}_{total}$ | Medium Risk  %, ${n/n}_{total}$ | High Risk  %, ${n/n}_{total}$ | Changed Categories  $n$ |
| Asia-Pacific | 8%, 1/12 | 0%, 0/9 | 70%, 21/30 | 29 |
| CCASAnet | 0%, 0/1 | 33%, 1/3 | 83%, 5/6 | 4 |
| Central Africa | NA | 100%, 17/17 | 100%, 3/3 | 0 |
| East Africa | 3%, 1/31 | 7%, 1/14 | 98%, 57/58 | 44 |
| NA-ACCORD | 67%, 34/51 | 33%, 1/3 | 11%, 1/9 | 27 |
| Southern Africa | 36%, 31/85 | 62%, 195/312 | 73%, 44/60 | 187 |
| West Africa | 100%, 1/1 | 0%, 0/2 | 7%, 1/14 | 15 |

***Table S7*** *This table describes the stability of each clinic in each flood risk category given the ranking variable is calculated at the upper bound of the 90% confidence interval from the final models. In each column the % is the number of clinics in the risk category from the upper bound ranking divided by the number of clinics in the risk category from the mean estimate ranking; fractions given following. A value of 100% indicates the same exact clinics appeared in that category both at the mean estimate and upper bound. A value of NA means no clinics were observed in that category based on the estimate mean. The last column indicates how many clinics changed categories when the ranking variable is calculated at the upper bound of the 90% confidence interval.*

As compared to the drought tables, the flood tables are less stable. There is more movement between categories across most regions. Interestingly, there is also more evidence of specific clinics moving jointly across categories of risk. For example, consider East Africa evaluated at the upper-bound of the 90% confidence interval. A full 90 clinics moved categories, and no category maintained more than 36% of its original (mean estimate) clinics. This suggests that clinics are moving in clumps -- which aligns with how the flood exposures are modeled. For each flood (as opposed to clinic), there is a singular displacement measure and duration that are measured at the *flood level*, that is, they aren't specific to each clinic. Hence, many of the rankings of the flood coefficients are tied, especially if those clinics are clustered and experienced the same flood exposures over the course of the period. Hence, in using the quantile cutoffs to classify the flood coefficients, we are picking up movements of clusters from one category to another. Additional clinic-specific flood metrics (such as soil moisture or topography around the clinic) could help refine these models and would change the risk quantiles. This is an area of future work for the authors currently. However, even though these models show less stable ranking results, they are still exploratory and indicative of changing risk across a landscape -- which is part of what we wished to convey.
